# Association between high-density lipoprotein cholesterol and lumbar bone mineral density in Chinese: a large cross-sectional study

**DOI:** 10.1186/s12944-024-02023-1

**Published:** 2024-01-24

**Authors:** Yongbing Sun, Xin Qi, Xuan Wang, Xinbei Lin, Yang Zhou, Yawei Du, Ao Liu, Xue Lv, Jing Zhou, Zhonglin Li, Xiaoling Wu, Zhi Zou, Michael Zhang, Jiadong Zhu, Feifei Shang, Yongli Li, Hao Li

**Affiliations:** 1 Department of Medical Imaging, People’s Hospital of Zhengzhou University, #7 Wei Wu Road, Zhengzhou, Henan 450003 China; 2 Department of Medical Imaging, Henan Provincial People’s Hospital, Xinxiang Medical College, Zhengzhou, Henan 450003 China; 3 Department of Medical Imaging, The Third Affiliated Hospital of Zhengzhou University, #7 Kungfu Street, Zhengzhou, Henan 450052 China; 4 Department of Medical Imaging, Henan Provincial People’s Hospital, Henan University People’s Hospital, Zhengzhou, Henan 450003 China; 5 Henan Provincial People’s Hospital, Zhengzhou, Henan 450003 China; 6 Department of Health Management, Chronic Health Management Laboratory, Henan Provincial People’s Hospital, Zhengzhou, Henan 450003 China; 7 Department of Nuclear Medicine, Henan Provincial People’s Hospital, Zhengzhou, Henan 450003 China; 8 Sevenoaks Health Management Center, Canada-Canada Institute of Health Engineering, University of Manitoba, Winnipeg, Canada; 9 Fuwaihua Central Vascular Disease Hospital, #1 Fuwai Avenue, Zhengzhou, Henan 451464 China

**Keywords:** Bone mineral density, High-density lipoprotein cholesterol, Osteoporosis, Cross-sectional study, Chinese

## Abstract

**Background:**

The association between lipid and bone metabolism, particularly the role of high-density lipoprotein cholesterol (HDL-C) in regulating bone mineral density (BMD), is of significant interest. Despite numerous studies, findings on this relationship remain inconclusive, especially since evidence from large, sexually diverse Chinese populations is sparse. This study, therefore, investigates the correlation between HDL-C and lumbar BMD in people of different genders using extensive population-based data from physical examinations conducted in China.

**Methods:**

Data from a cross-sectional survey involving 20,351 individuals aged > = 20 years drawn from medical records of health check-ups at the Health Management Centre of the Henan Provincial People’s Hospital formed the basis of this study. The primary objective was to determine the correlation between HDL-C levels and lumbar BMD across genders. The analysis methodology included demographic data analysis, one-way ANOVA, subgroup analyses, multifactorial regression equations, smoothed curve fitting, and threshold and saturation effect analyses.

**Results:**

Multifactorial regression analysis revealed a significant inverse relationship between HDL-C levels and lumbar BMD in both sexes, controlling for potential confounders (Male: *β* = -8.77, 95% CI -11.65 to -5.88, *P* < 0.001; Female: *β* = -4.77, 95% CI -8.63 to -0.90, *P* = 0.015). Subgroup and threshold saturation effect analyses indicated a stronger association in males, showing that increased HDL-C correlates with reduced lumbar BMD irrespective of age and body mass index (BMI). The most significant effect was observed in males with BMI > 28 kg/m^2^ and HDL-C > 1.45 mmol/L and in females with a BMI between 24 and 28 kg/m^2^.

**Conclusion:**

Elevated HDL-C is associated with decreased bone mass, particularly in obese males. These findings indicate that individuals with high HDL-C levels should receive careful clinical monitoring to mitigate osteoporosis risk.

**Trial registration:**

The research protocol received ethics approval from the Ethics Committee at Beijing Jishuitan Hospital, in conformity with the Declaration of Helsinki guidelines (No. 2015-12-02). These data are a contribution of the China Health Quantitative CT Big Data Research team, registered at clinicaltrials.gov (code: NCT03699228).

## Introduction

Osteoporosis (OP) is a systemic skeletal condition characterized by reduced bone density and deterioration of bone tissue microarchitecture, resulting in increased bone fragility and a higher fracture risk [1]. It affects approximately 200 million people worldwide [2]. According to 2019 survey data, OP exhibits a high prevalence in China, affecting 6.46% of males and 29.13% of females over the age of 50 [3]. With the increasing aging population, the societal significance of OP can no longer be overlooked, making it a global public health concern. Reduced bone density serves as a diagnostic indicator for OP, with lumbar bone mineral density (BMD) being one of its commonly utilized measures [4].

In recent years, the interplay between lipid metabolism and bone metabolism has attracted significant attention. Studies have indicated that individuals with OP and decreased bone mass often exhibit abnormalities in lipid metabolism, particularly in serum high-density lipoprotein cholesterol metabolism [5], with a specific focus on serum HDL-C abnormalities [6]. Nevertheless, prior investigations have highlighted the influence of numerous confounding factors on the relationship between HDL-C and BMD [7, 8]. The variation in findings can be attributed to small sample sizes, regional disparities, and differences in control for confounding factors. Consequently, it is crucial to examine the correlation between HDL-C and lumbar BMD using a large, homogeneous dataset.

Lipids play a pivotal role in various metabolic disorders [9]. HDL-C, which is responsible for transporting excess cholesterol from peripheral sites to the liver, contributes to reducing the risk of cardiovascular diseases [10]. However, recent research has indicated that elevated HDL-C levels in adults ( > = 135 mg/dl in females and > = 116 mg/dl in males) may lead to increased all-cause mortality [11]. Another extensive study demonstrated a positive association between HDL-C levels and high mortality rates [12]. These findings have prompted a reevaluation of HDL-C’s role in metabolic processes. The relationship between HDL-C and bone metabolism has long been a subject of intrigue. Jiang et al. identified elevated HDL-C levels as an independent risk factor for bone loss in both men and women based on comprehensive multicenter data collected in China [13]. However, some studies have reported positive correlations [14], while others have found no association [15] between HDL-C and BMD. Consequently, the link between HDL-C and BMD remains a subject of significant controversy. Investigating the effect of HDL-C on BMD could establish a new theoretical basis for preventing and managing bone loss, as well as addressing the emergence of OP, which holds substantial clinical relevance.

Therefore, the present study acquired data from five consecutive years of physical examinations at the Health Screening Center of Henan Provincial People’s Hospital. The primary objective was to investigate the relationship between HDL-C and lumbar BMD in Chinese populations of different sexes. Multiple analytical methods were employed, and various factors potentially influencing lipid and bone metabolism were included as covariates to enhance the study’s robustness.

## Materials and methods

### Participants and inclusion criterion

The data for this study were sourced from the medical records of individuals undergoing medical examinations at the Health Management Center of Henan Provincial People’s Hospital, spanning from February 2018 to February 2023. This dataset is an integral component of the China Health Quantitative CT Big Data Research Project Group and has been duly registered with clinicaltrials.gov (code: NCT03699228).

The inclusion criteria for participants included the following: (1) individuals aged between 20 and 80 years, (2) those possessing comprehensive demographic information, and (3) individuals who underwent routine low-dose chest CT scans and lipid assessments. Exclusion criteria were as follows: (1) a history of any form of cancer, (2) prior or current endocrine-related ailments, (3) previous or existing metabolic conditions associated with the liver or kidneys, and (4) previous or ongoing usage of medications designed for OP prevention and blood lipid regulation. Thorough data collection, including age, gender, ethnicity, medical history, and medication history, was carried out on all participants by trained research personnel through on-site surveys.

**Fig. 1:**
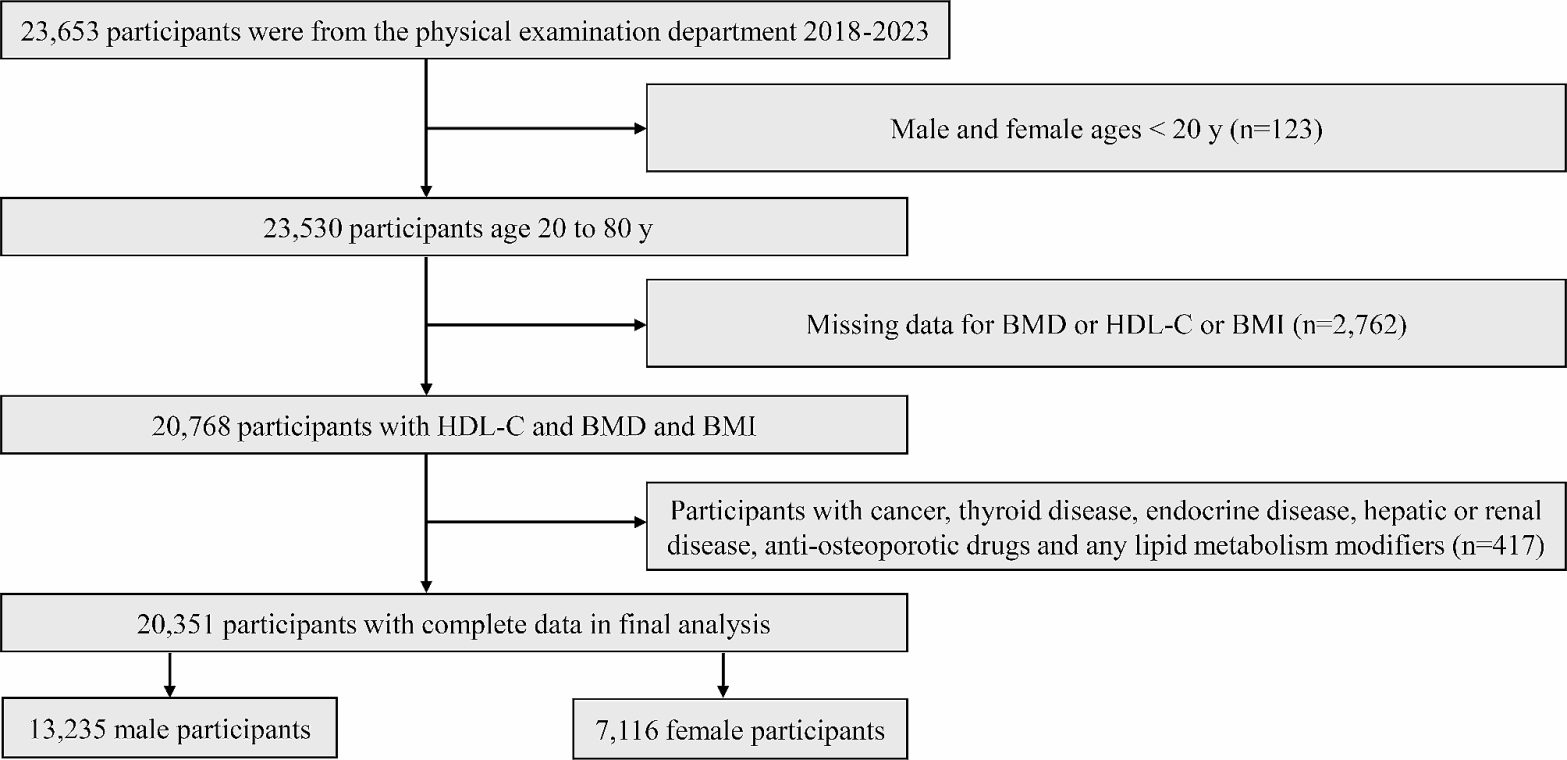
Flowchart of participants selection

Initially, a total of 23,653 participants were enrolled in this research initiative. However, 123 subjects were deemed ineligible due to age constraints, while 2,762 participants were excluded due to the absence of lumbar BMD, HDL-C, or body mass index (BMI) data. Additionally, 417 participants were disqualified owing to disparate medical histories that failed to meet the stipulated inclusion criteria. Consequently, the final cohort for this study comprised 20,351 participants, including 13,235 males and 7,116 females. The process of subject screening is visually depicted in Fig. 1.

### Methods of research

To ensure data accuracy and impartiality, all researchers underwent standardized training before conducting the surveys. A uniform questionnaire was employed to collect essential information from the participants. This questionnaire included details of the participants’ medical history, including any prior or current occurrences of various cancers, liver and kidney ailments, thyroid and endocrine disorders, and the utilization of anti-osteoporotic medications and lipid metabolism modifiers. The completion of the questionnaire was followed by data compilation, summary, and verification.

Subsequently, following a 12 h fasting period, the participants’ height, weight, and blood pressure were measured in the morning by the research personnel. Each participant underwent two measurements, and the average of these two measurements was calculated to minimize potential errors. BMI was defined as the quotient of weight divided by height squared (kg/m^2^).

### Measurements in the laboratory

Fasting blood samples were systematically collected at 8 am to measure HDL-C and various other laboratory parameters, which included total cholesterol (TC), low-density lipoprotein cholesterol (LDL-C), triglycerides (TG), total protein (TP), albumin (Alb), hemoglobin (Hb), globulin, total bilirubin (TB), direct bilirubin, indirect bilirubin, alanine aminotransferase (ALT), aspartate transaminase (AST), glutamine transaminase, alkaline phosphatase (ALP), fasting blood glucose (FBG), blood potassium, blood chloride, blood calcium, blood creatinine, uric acid (UA), blood phosphorus, and glycosylated hemoglobin (GH). Blood glucose and lipid levels were assessed using an Olympus® AU 5400 automated biochemistry analyzer (Olympus Corporation, Japan, Shizuoka Prefecture). The remaining indicators were evaluated using standard laboratory techniques.

### BMD measurement

Low-dose chest CT scans constituted a component of the participants’ routine health assessments, and measurements of BMD were derived from standard examination images, thus obviating any additional radiation exposure for the participants. The CT scans for each participant were executed using uniform parameters.

Volumetric BMD (vBMD) measurements were conducted employing Mindways quantitative computed tomography (QCT) Pro (Mindways Software, Inc., Austin, TX, USA). Specifically, trabecular vBMD (mg/cm^3^) of the lumbar vertebrae (L1-L2) was assessed utilizing asynchronous BMD calibration and QCT Pro analysis (Mindways Software Inc., Austin, United States). Each vertebra underwent three measurements, and the resultant average of these three measurements was deemed the BMD for each individual vertebra. The final BMD value was computed as the average of the measurements for both vertebrae. Radiologists who had received specialized training conducted all analyses using QCT software. It is worth noting that a previously published study validated the suitability of this approach for Chinese individuals [16].

To ensure consistent quality control throughout the research, routine calibration and cross-comparisons among systems were upheld, employing the European Spinal Prosthesis-145 (ESP-145) as a benchmark.

### Variables

In this study, BMD was employed as the dependent variable, with HDL-C serving as the independent variable. The covariates taken into consideration included ethnicity, marital status, age, BMI, systolic blood pressure (SBP), diastolic blood pressure (DBP), TC, LDL-C, TG, TP, Alb, Hb, globulin, TB, direct bilirubin, indirect bilirubin, ALT, AST, glutamine aminotransferase, ALP, FBG, potassium, chloride, calcium, creatinine, UA, phosphorus, and GH.

### Statistical analysis

The statistical analysis was carried out using two software packages, namely, R and Empower. All data were tested for normality, with means ± standard deviations indicating normally distributed continuous variables and medians indicating non-normally distributed continuous variables. In this assessment, we initially characterized the participants’ traits by presenting means or medians with quartiles for continuous variables and proportions for categorical variables. To address significant variations within the dataset, both chi-square tests and variance estimation were employed. The investigation of the correlation between HDL-C levels and lumbar BMD was conducted using a multiple linear regression model. Independent variables were scrutinized through a stepwise regression method, with the exclusion of those exhibiting severe square collinearity. Furthermore, a multivariate linear regression model was applied to perform subgroup analyses of the linear association between HDL-C and lumbar BMD across different sex groups, categorized by age and BMI. To discern potential nonlinear relationships between HDL-C levels and lumbar BMD, a smooth curve fitting technique and a generalized additive model were utilized. Objective calculations were employed to identify inflection points in the correlation between HDL-C and lumbar BMD, and a two-stage linear regression model was established on either side of these inflection points. Statistical significance was established at a two-tailed *P* < 0.05. The graphic representation depicting the distribution of serum HDL-C levels was generated using Origin software (Origin Lab, USA, version 2021b).

## Result

### Baseline characteristics of participants

**Fig. 2:**
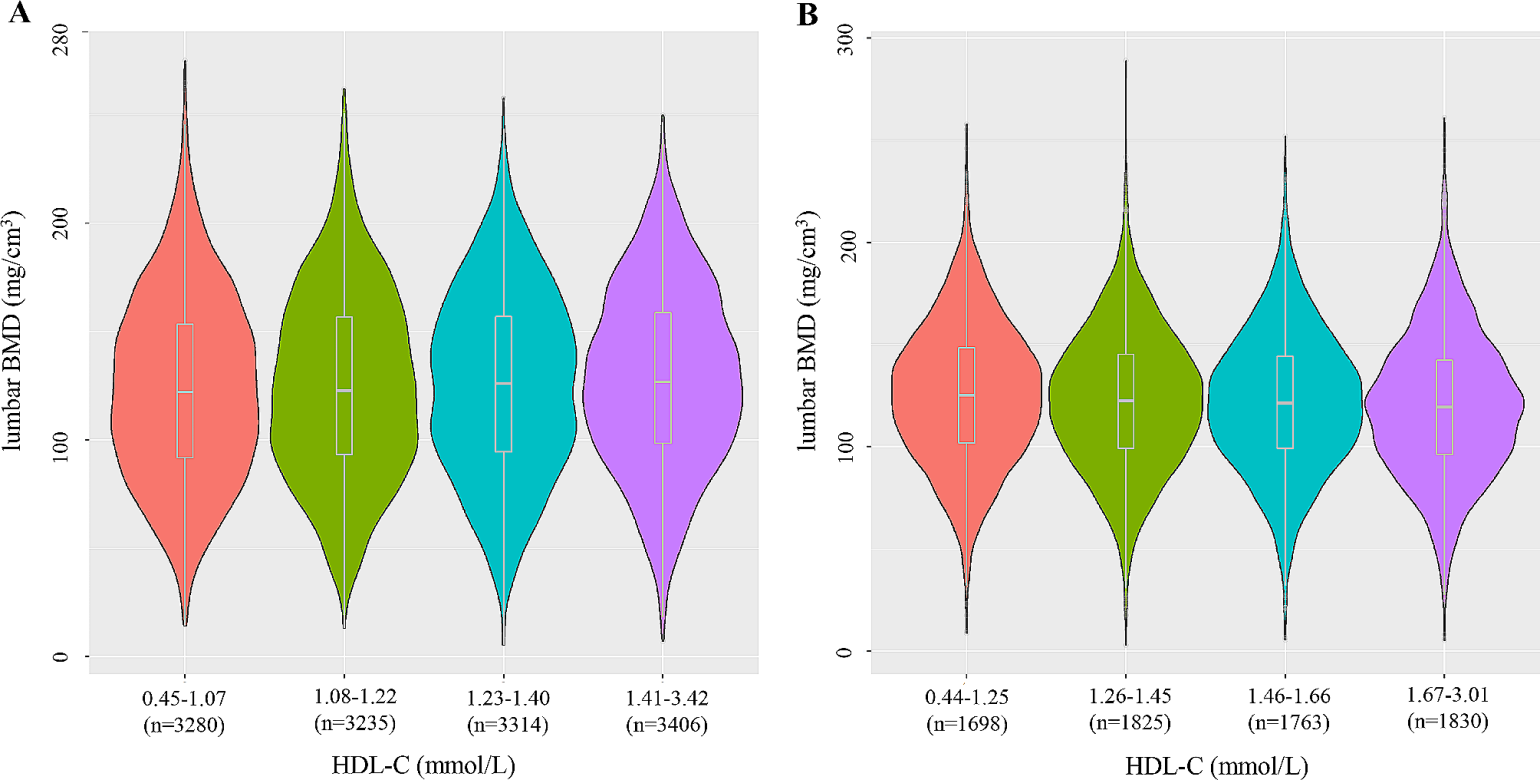
The distribution of HDL-C in different gender groups was quartered. (**A**) males; (**B**) females; HDL-C, high-density lipoprotein cholesterol, BMD, bone mineral density

A total of 20,351 individuals participated in this study, comprising 13,235 men and 7,116 women. The HDL-C levels were categorized into quartiles based on sex, with the following ranges: (male: Q1: 0.45–1.07; Q2: 1.08–1.22; Q3: 1.23–1.40; Q4: 1.41–3.42. Female: Q1: 0.44–1.25; Q2: 1.26–1.45; Q3: 1.46–1.66; Q4: 1.67–3.01). The distribution of BMD across HDL-C quartiles is depicted in Fig. 2.

Tables 1 and 2 illustrate substantial variations in baseline characteristics among HDL-C quartiles in both men and women. Men in the Q4 HDL-C group tended to be older, married, and exhibited lower height, weight, BMI, DBP, TG, Hb, ALT, ALP, FBG, UA, GH, and BMD and higher TC, LDL-C, Alb, TB, direct bilirubin, and indirect bilirubin than those in the other subgroups.

**Table 1:** Characteristics of the male study population

Male (*n* = 13,235)					
HDL-C (mmol/L)	Q1(0.45–1.07)	Q2(1.08–1.22)	Q3(1.23–1.40)	Q4(1.41–3.42)	*P*-value
Age (year), n (%)					< 0.001^***^
<= 45	886 (27.01)	748 (23.12)	732 (22.09)	599 (17.59)	
> 45	2,394 (72.99)	2,487 (76.88)	2,582 (77.92)	2,807 (82.42)	
Nationality n (%)					0.452
Han nationality	3,252 (99.15)	3,212 (99.29)	3,296 (99.46)	3,385 (99.38)	
non-Han nationality	28 (0.85)	23 (0.71%)	18 (0.54)	21 (0.62)	
Marital status, n (%)					0.035^*^
Not married	87 (2.65)	103 (3.18)	115 (3.47)	133 (3.91)	
Married	3,193 (97.35)	3,132 (96.82)	3,199 (96.53)	3,273 (96.09)	
High (m)	1.72 ± 0.06	1.72 ± 0.06	1.72 ± 0.05	1.72 ± 0.05	< 0.001^***^
Weight (kg)	78.73 ± 10.43	76.68 ± 9.84	74.89 ± 10.04	71.22 ± 9.74	< 0.001^***^
BMI (kg/m^2^), n (%)					< 0.001^***^
<= 24	623 (18.99)	802 (24.79)	1,130 (34.10)	1,666 (48.91)	
> 24, <= 28	1,757 (53.57)	1,778 (54.96)	1,641 (49.52)	1,430 (41.99)	
> 28	900 (27.44)	655 (20.25)	543 (16.39)	310 (9.10)	
DBP (mmHg)	79.02 ± 11.89	78.74 ± 11.70	78.80 ± 11.77	78.21 ± 11.79	0.036^*^
SBP (mmHg)	130.84 ± 17.80	131.13 ± 18.04	131.29 ± 18.01	130.79 ± 18.81	0.636
TC (mmol/L)	4.49 ± 1.01	4.72 ± 0.93	4.89 ± 0.95	5.08 ± 0.93	< 0.001^***^
LDL-C (mmol/L)	2.64 ± 0.75	2.88 ± 0.76	2.96 ± 0.81	2.98 ± 0.81	< 0.001^***^
TG (mmol/L)	2.84 ± 2.26	2.00 ± 1.14	1.70 ± 0.89	1.36 ± 0.67	< 0.001^***v^
TP (g/L)	71.28 ± 4.38	71.49 ± 4.02	71.49 ± 3.95	71.51 ± 4.04	0.072
Alb (g/L)	45.31 ± 2.77	45.41 ± 2.71	45.47 ± 2.69	45.50 ± 2.76	0.029^*^
Hb (g/L)	151.41 ± 11.97	151.47 ± 10.74	151.28 ± 10.74	149.89 ± 10.96	< 0.001^***^
Globulin (g/L)	25.97 ± 3.88	26.08 ± 3.43	26.03 ± 3.27	26.01 ± 3.32	0.622
TB (µmol/L)	12.70 ± 5.74	13.39 ± 5.47	13.66 ± 5.49	14.62 ± 5.89	< 0.001^***^
Direct bilirubin (umol/L)	3.13 ± 1.40	3.19 ± 1.35	3.18 ± 1.14	3.31 ± 1.23	< 0.001^***^
Indirect bilirubin (µmol/L)	9.57 ± 4.78	10.21 ± 4.57	10.49 ± 4.67	11.31 ± 5.01	< 0.001^***^
ALT (U/L)	24.00 (17.70-33.93)	22.30 (16.80–31.30)	21.40 (15.90–29.70)	19.35 (14.90–26.40)	< 0.001^***^
AST (U/L)	23.16 ± 10.28	23.28 ± 13.68	22.68 ± 8.89	23.09 ± 11.33	0.145
Transglutaminase (U/L)	30.00 (22.20-43.93)	28.70 (20.80–41.80)	27.60 (20.30-42.18)	25.70 (19.00-39.38)	0.173
ALP (U/L)	71.18 ± 18.46	68.87 ± 17.94	67.63 ± 17.97	65.11 ± 17.00	< 0.001^***^
FBG (mmol/L)	5.75 ± 1.79	5.56 ± 1.49	5.43 ± 1.30	5.35 ± 1.19	< 0.001^***^
Serum potassium (mmol/L)	4.23 ± 0.09	4.23 ± 0.09	4.23 ± 0.09	4.23 ± 0.09	0.961
Serum chlorine (mmol/L)	105.09 ± 0.67	105.09 ± 0.65	105.08 ± 0.66	105.06 ± 0.68	0.235
Serum calcium (mmol/L)	2.34 ± 0.03	2.34 ± 0.02	2.34 ± 0.03	2.34 ± 0.02	0.115
Serum creatinine (umol/L)	74.61 ± 23.21	75.57 ± 30.41	75.31 ± 21.94	75.07 ± 13.99	0.379
UA (umol/L)	384.52 ± 83.62	378.08 ± 82.89	369.78 ± 76.78	356.35 ± 75.96	< 0.001^***^
Serum phosphorus (mmol/L)	1.04 ± 0.03	1.04 ± 0.03	1.04 ± 0.02	1.04 ± 0.02	0.091
GH (%)	6.09 ± 1.01	5.98 ± 0.84	5.92 ± 0.74	5.86 ± 0.71	< 0.001***
BMD (mg/cm^3^)	125.42 ± 34.04	122.50 ± 33.65	121.71 ± 33.74	119.91 ± 34.67	< 0.001***

HDL-C, high-density lipoprotein cholesterol; BMI, body mass index; DBP, diastolic blood pressure; SBP, systolic blood pressure; TC, total cholesterol; LDL-C, low-density lipoprotein cholesterol; TG, triglycerides; TP, total protein; Alb, albumin; Hb, Hemoglobin; TB, total bilirubin; ALT, alanine aminotransferase; AST, aspartate aminotransferase; ALP, alkaline phosphatase; FBG, fasting blood glucose; UA, uric acid; GH, glycosylated hemoglobin; BMD, bone mineral density; n, number of subjects; %, weighted percentage

^*^P < 0.05, ^**^P < 0.01, ^***^P < 0.001

**Table 2:** Characteristics of the female study population

Female (*n* = 7,116)					
HDL-C (mmol/L)	Q1(0.44–1.25)	Q2(1.26–1.45)	Q3(1.46–1.66)	Q4(1.67–3.01)	*P*-value
Age (year), n (%)					0.040^*^
<= 45	299 (17.61)	379 (20.77)	357 (20.25)	387 (21.15)	
> 45	1,399 (82.39)	1,446 (79.23)	1,406 (79.75)	1,443 (78.85)	
Nationality n (%)					0.584
Han nationality	1,673 (98.53)	1,796 (98.41)	1,728 (98.02)	1,803 (98.53)	
non-Han nationality	25 (1.47)	29 (1.59)	35 (1.98)	27 (1.47)	
Marital status n (%)					< 0.001^***^
Not married	24 (1.41)	33 (1.81)	50 (2.84)	64 (3.50)	
Married	1,674 (98.59)	1,792 (98.19)	1,713 (97.16)	1,766 (96.50)	
High (m)	1.59 ± 0.05	1.60 ± 0.06	1.59 ± 0.05	1.60 ± 0.05	< 0.001^***^
Weight (kg)	63.14 ± 8.58	61.63 ± 8.09	59.16 ± 7.53	56.91 ± 7.19	< 0.001^***^
BMI (kg/m^2^), n (%)					< 0.001^***^
<= 24	688 (40.52)	925 (50.69)	1131 (64.15)	1391 (76.01)	
> 24, <= 28	756 (44.52)	698 (38.25)	508 (28.81)	386 (21.09)	
> 28	254 (14.96)	202 (11.07)	124 (7.03)	53 (2.90)	
DBP (mmHg)	73.47 ± 12.02	72.98 ± 11.64	71.31 ± 11.07	70.80 ± 11.18	< 0.001^***^
SBP (mmHg)	128.98 ± 21.50	127.55 ± 21.15	124.28 ± 19.76	122.66 ± 19.45	< 0.001^***^
TC (mmol/L)	4.75 ± 0.96	5.03 ± 0.93	5.18 ± 0.96	5.43 ± 0.91	< 0.001^***^
LDL-C (mmol/L)	2.83 ± 0.74	3.00 ± 0.79	2.98 ± 0.82	2.89 ± 0.78	< 0.001^***^
TG (mmol/L)	2.11 ± 1.19	1.52 ± 0.71	1.30 ± 0.54	1.10 ± 0.594	< 0.001^***^
TP (g/L)	71.80 ± 4.104	71.74 ± 3.85	71.93 ± 3.93	72.15 ± 3.99	0.011^*^
Alb (g/L)	44.33 ± 2.56	44.66 ± 2.43	44.77 ± 2.48	45.08 ± 2.51	< 0.001^***^
Hb (g/L)	130.01 ± 12.47	130.51 ± 11.96	131.12 ± 10.74	130.06 ± 11.15	0.015^*^
Globulin (g/L)	27.47 ± 3.72	27.07 ± 3.34	27.16 ± 3.34	27.06 ± 3.20	< 0.001^***^
TB (µmol/L)	10.11 ± 3.93	10.55 ± 4.14	10.84 ± 4.33	11.07 ± 4.10	< 0.001^***^
Direct bilirubin (umol/L)	2.36 ± 0.83	2.39 ± 0.83	2.44 ± 0.86	2.43 ± 0.84	0.018^*^
Indirect bilirubin (µmol/L)	7.74 ± 3.43	8.16 ± 3.60	8.39 ± 3.80	8.64 ± 3.58	< 0.001^***^
ALT (U/L)	17.50 (13.20–24.20)	16.30 (12.30–21.70)	15.800 (12.20-20.95)	15.20 (11.90-20.57)	< 0.001^***^
AST (U/L)	21.74 ± 10.84	20.92 ± 8.25	21.35 ± 8.69	21.87 ± 10.72	0.014^*^
Transglutaminase (U/L)	19.40 (14.80–26.70)	17.30 (13.60–23.30)	16.50 (13.10–22.30)	15.50 (12.70–20.40)	< 0.001^***^
ALP (U/L)	72.49 ± 22.25	69.82 ± 24.49	68.74 ± 22.06	64.56 ± 19.37	< 0.001^***^
FBG (mmol/L)	5.37 ± 1.38	5.16 ± 1.03	5.10 ± 1.04	5.00 ± 0.94	< 0.001^***^
Serum potassium (mmol/L)	4.19 ± 0.06	4.19 ± 0.07	4.19 ± 0.07	4.19 ± 0.08	0.413
Serum chlorine (mmol/L)	105.80 ± 0.53	105.82 ± 0.54	105.83 ± 0.57	105.80 ± 0.61	0.321
Serum calcium (mmol/L)	2.35 ± 0.02	2.35 ± 0.02	2.35 ± 0.02	2.35 ± 0.02	0.066
Serum creatinine (umol/L)	55.98 ± 11.01	56.71 ± 10.73	57.39 ± 10.66	58.38 ± 9.18	< 0.001^***^
UA (umol/L)	295.53 ± 67.99	283.34 ± 63.21	275.95 ± 61.92	267.43 ± 57.24	< 0.001^***^
Serum phosphorus (mmol/L)	1.17 ± 0.03	1.17 ± 0.02	1.17 ± 0.02	1.17 ± 0.03	0.256
GH (%)	5.96 ± 0.77	5.84 ± 0.64	5.78 ± 0.59	5.76 ± 0.58	< 0.001^***^
BMD (mg/cm^3^)	123.94 ± 42.86	124.83 ± 43.29	125.76 ± 42.65	128.22 ± 41.73	0.018^*^

HDL-C, high-density lipoprotein cholesterol; BMI, body mass index; DBP, diastolic blood pressure; SBP, systolic blood pressure; TC, total cholesterol; LDL-C, low-density lipoprotein cholesterol; TG, triglycerides; TP, total protein; Alb, albumin; Hb, Hemoglobin; TB, total bilirubin; ALT, alanine aminotransferase; AST, aspartate aminotransferase; ALP, alkaline phosphatase; FBG, fasting blood glucose; UA, uric acid; GH, glycosylated hemoglobin; BMD, bone mineral density; n, number of subjects; %, weighted percentage

^*^P < 0.05, ^**^P < 0.01, ^***^P < 0.001

**Fig. 3:**
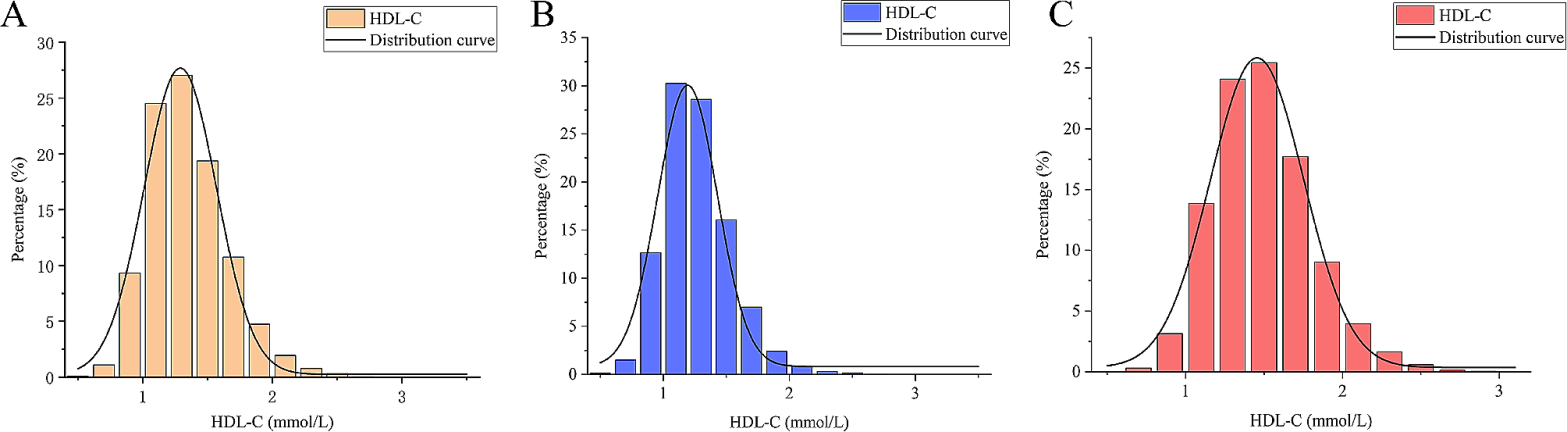
Distribution histogram of HDL-C. (**A**) all participants; (**B**) all males; (**C**) all females; HDL-C, high-density lipoprotein cholesterol

In contrast, women in the Q4 HDL-C group tended to be older, married, taller, and had higher TC, TP, Alb, TB, direct bilirubin, indirect bilirubin, serum creatinine and lumbar BMD, whereas body weight, BMI, DBP, SBP, LDL-C, TG, Hb, globulin, ALT, AST, transglutaminase, ALP, FBG and GH. The distribution of HDL-C among the participants is visualized in Fig. 3.

### Univariate analysis

The results of the univariate analysis demonstrated that in the male cohort, lumbar BMD exhibited a negative correlation with age > 45, marital status, SBP, globulin, direct bilirubin, ALP, FBG, and GH. Conversely, most of the other factors demonstrated a positive correlation (Table 3). Conversely, in the female cohort, lumbar BMD displayed a positive correlation with height and Alb. In contrast, the remaining significant factors exhibited a negative correlation with lumbar BMD (Table 4).

**Table 3:** Results of univariate analysis of male

	Statistics	Effect size (β)	*P* value
Male (*n* = 13,235)			
Age (year), n (%)			
<= 45	2,965 (22.40)	Reference	
> 45	10,270 (77.60)	-34.85 (-36.11, -33.59)	< 0.001^***^
Nationality, n (%)			
Han nationality	13,145 (99.32)	Reference	
non-Han nationality	90 (0.68)	3.950 (-3.12, 11.02)	0.273
Marital status n (%)			
Not married	438 (3.31)	Reference	
Married	12,797 (96.69)	-26.26 (-29.48, -23.05)	< 0.001^***^
High (m)	1.72 ± 0.05	66.15 (56.06, 76.25)	< 0.001^***^
Weight (kg)	75.33 ± 10.39	0.39 (0.33, 0.44)	< 0.001^***^
BMI (kg/m^2^), n (%)			
<= 24	4,221 (31.89)	Reference	
> 24, <= 28	6,606 (49.91)	2.55 (1.23, 3.86)	< 0.001^***^
> 28	2,408 (18.19)	6.47 (4.77, 8.17)	< 0.001^***^
DBP (mmHg)	78.69 ± 11.79	0.05 (0.01, 0.11)	0.020^*^
SBP (mmHg)	131.01 ± 18.17	-0.29 (-0.33, -0.26)	< 0.001^***^
TC (mmol/L)	4.80 ± 0.97	3.21 (2.62, 3.80)	< 0.001^***^
LDL-C (mmol/L)	2.84 ± 0.79	4.04 (3.31, 4.77)	< 0.001^***^
TG (mmol/L)	1.96 ± 1.48	1.52 (1.13, 1.91)	< 0.001^***^
TP (g/L)	71.44 ± 4.10	0.57 (0.43, 0.71)	< 0.001^***^
Alb (g/L)	45.42 ± 2.73	3.10 (2.91, 3.31)	< 0.001^***^
Hb (g/L)	151.00 ± 11.13	0.57 (0.52, 0.62)	< 0.001^***^
Globulin (g/L)	26.02 ± 3.48	-1.12 (-1.29, -0.96)	< 0.001^***^
TB (µmol/L)	13.60 ± 5.69	0.02 (-0.08, 0.12)	0.665
Direct bilirubin (umol/L)	3.20 ± 1.28	-1.03 (-1.48, -0.57)	< 0.001^***^
Indirect bilirubin (µmol/L)	10.40 ± 4.80	0.10 (-0.01, 0.22)	0.087
ALT (U/L)	26.26 ± 18.83	0.22 (0.19, 0.25)	< 0.001^***^
AST (U/L)	23.05 ± 11.17	0.09 (0.04, 0.14)	< 0.001^***^
Transglutaminase (U/L)	37.91 ± 37.95	0.04 (0.03, 0.06)	< 0.001^***^
ALP (U/L)	68.16 ± 17.97	-0.14 (-0.17, -0.11)	< 0.001^***^
FBG (mmol/L)	5.51 ± 1.46	-2.26 (-2.65, -1.87)	< 0.001^***^
Serum potassium (mmol/L)	4.23 ± 0.09	-1.87 (-8.34, 4.59)	0.569
Serum chlorine (mmol/L)	105.08 ± 0.66	-0.50 (-1.38, 0.37)	0.257
Serum calcium (mmol/L)	2.34 ± 0.02	16.63 (-7.39, 40.65)	0.174
Serum creatinine (umol/L)	75.13 ± 23.03	-0.02 (-0.04, 0.01)	0.170
UA (umol/L)	372.00 ± 80.52	0.05 (0.04, 0.06)	< 0.001^***^
Serum phosphorus (mmol/L)	1.04 ± 0.03	2.94 (-18.83, 24.72)	0.791
GH (%)	5.96 ± 0.84	-5.44 (-6.13, -4.75)	< 0.001^***^

HDL-C, high-density lipoprotein cholesterol; BMI, body mass index; DBP, diastolic blood pressure; SBP, systolic blood pressure; TC, total cholesterol; LDL-C, low-density lipoprotein cholesterol; TG, triglycerides; TP, total protein; Alb, albumin; Hb, Hemoglobin; TB, total bilirubin; ALT, alanine aminotransferase; AST, aspartate aminotransferase; ALP, alkaline phosphatase; FBG, fasting blood glucose; UA, uric acid; GH, glycosylated hemoglobin; BMD, bone mineral density; n, number of subjects; %, weighted percentage

^*^P < 0.05, ^**^P < 0.01, ^***^P < 0.001

**Table 4:** Results of univariate analysis of female

	Statistics	Effect size (β)	*P* value
Female (*n* = 7,116)			
Age (year), n (%)			
<= 45	1,422 (19.98)	Reference	
> 45	5,694 (80.02)	-54.00 (-56.14, -51.86)	< 0.001^***^
Nationality n (%)			
Han nationality	7,000 (98.37)	Reference	
non-Han nationality	116 (1.63)	3.828 (-3.99, 11.65)	0.337
Marital status n (%)			
Not married	171 (2.40)	Reference	
Married	6,945 (97.59)	-42.87 (-49.27, -36.48)	< 0.001^***^
High (m)	1.59 ± 0.05	164.9 (147.16, 182.81)	< 0.001^***^
Weight (kg)	60.16 ± 8.20	-0.21 (-0.34, -0.09)	< 0.001^***^
BMI (kg/m^2^), n (%)			
< 24	4,135 (58.11)	Reference	
>= 24, < 28	2,348 (32.99)	-10.05(-12.19, -7.92)	< 0.001^***^
>= 28	633 (8.90)	-17.78 (-21.31, -14.24)	< 0.001^***^
DBP (mmHg)	72.12 ± 11.53	-0.56 (-0.65, -0.48)	< 0.001^***^
SBP (mmHg)	125.82 ± 20.62	-0.77 (-0.82, -0.73)	< 0.001^***^
TC (mmol/L)	5.10 ± 0.97	-5.70 (-6.71, -4.69)	< 0.001^***^
LDL-C (mmol/L)	2.93 ± 0.78	-5.45 (-6.71, -4.21)	< 0.001^***^
TG (mmol/L)	1.50 ± 0.87	-7.90 (-9.02, -6.78)	< 0.001^***^
TP (g/L)	71.91 ± 3.96	-0.03 (-0.28, 0.21)	0.784
Alb (g/L)	44.72 ± 2.51	1.70 (1.31, 2.09)	< 0.001^***^
Hb (g/L)	130.43 ± 11.60	-0.54 (-0.62, -0.45)	< 0.001^***^
Globulin (g/L)	27.19 ± 3.41	-0.96 (-1.25, -0.67)	< 0.001^***^
TB (µmol/L)	10.65 ± 4.14	-0.30 (-0.54, -0.06)	0.012^*^
Direct bilirubin (umol/L)	2.41 ± 0.84	-2.78 (-3.95, -1.61)	< 0.001^***^
Indirect bilirubin (µmol/L)	8.24 ± 3.62	-0.24 (-0.52, 0.02)	0.076
ALT (U/L)	19.44 ± 14.25	-0.23 (-0.30, -0.16)	< 0.001^***^
AST (U/L)	21.47 ± 9.69	-0.64 (-0.74, -0.54)	< 0.001^***^
Transglutaminase (U/L)	21.76 ± 20.36	-0.16 (-0.21, -0.11)	< 0.001^***^
ALP (U/L)	68.84 ± 22.29	-0.69 (-0.73, -0.65)	< 0.001^***^
FBG (mmol/L)	5.16 ± 1.11	-7.36 (-8.24, -6.49)	< 0.001^***^
Serum potassium (mmol/L)	4.19 ± 0.07	-9.35 (-22.73, 4.04)	0.171
Serum chlorine (mmol/L)	105.82 ± 0.56	-0.01 (-1.75, 1.75)	0.998
Serum calcium (mmol/L)	2.35 ± 0.02	-101.74 (-145.90, -57.59)	< 0.001^***^
Serum creatinine (umol/L)	57.14 ± 10.44	-0.50 (-0.60, -0.41)	< 0.001^***^
UA (umol/L)	280.33 ± 63.43	-0.08 (-0.10, -0.07)	< 0.001^***^
Serum phosphorus (mmol/L)	1.17 ± 0.03	-16.64 (-52.59, 19.31)	0.364
GH (%)	5.84 ± 0.65	-14.48 (-15.96, -13.01)	< 0.001^***^

HDL-C, high-density lipoprotein cholesterol; BMI, body mass index; DBP, diastolic blood pressure; SBP, systolic blood pressure; TC, total cholesterol; LDL-C, low-density lipoprotein cholesterol; TG, triglycerides; TP, total protein; Alb, albumin; Hb, Hemoglobin; TB, total bilirubin; ALT, alanine aminotransferase; AST, aspartate aminotransferase; ALP, alkaline phosphatase; FBG, fasting blood glucose; UA, uric acid; GH, glycosylated hemoglobin; BMD, bone mineral density; n, number of subjects; %, weighted percentage

^*^P < 0.05, ^**^P < 0.01, ^***^P < 0.001

### Relationship between HDL-C and lumbar BMD

The results of the multiple regression analysis, as presented in Table 5, indicated that in the male cohort, there was a negative correlation with lumbar BMD in the unadjusted model (*β* = -7.03, 95% CI -9.18 to -4.84, *P* < 0.001). After adjusting for covariates, this negative correlation remained highly significant, with significant associations observed in both Model I (*β* = -7.33, 95% CI -9.46 to -5.19, *P* < 0.001) and Model II (*β* = -8.77, 95% CI -11.65 to -5.88, *P* < 0.001). The negative correlation persisted even after the conversion of HDL-C from a continuous variable to quartiles in all models, except for participants in Q4 in Model II.

**Table 5:** Relationship between HDL-C and lumbar BMD

	Crude Model		Model I		Model II	
	β (95%CI)	*P* value	β (95%CI)	*P* value	β (95%CI)	*P* value
**Male**						
HDL-C (mmol/L)	-7.03 (-9.18, -4.87)	< 0.001^***^	-7.33 (-9.46, -5.19)	< 0.001^***^	-8.77 (-11.65, -5.88)	< 0.001^***^
Q1	Reference		Reference		Reference	
Q2	-2.92 (-4.57, -1.27)	0.001^**^	-3.05 (-4.69, -1.42)	< 0.001^***^	-2.08 (-3.66, -0.50)	0.009^**^
Q3	-3.71 (-5.36, -2.07)	< 0.001^***^	-3.92 (-5.55, -2.29)	< 0.001^***^	-2.22 (-4.21, -0.23)	0.029^*^
Q4	-5.51 (-7.14, -3.88)	< 0.001^***^	-5.83 (-7.45, -4.22)	< 0.001^***^	-2.32 (-5.32, 0.67)	0.129
*P* for trend		< 0.001^***^		< 0.001^***^		0.082
**Female**						
HDL-C (mmol/L)	4.19 (1.01, 7.36)	0.009^**^	-1.96 (-4.11, 0.18)	0.073	-4.77 (-8.63, -0.90)	0.015^*^
Q1	Reference		Reference		Reference	
Q2	0.89 (-1.93, 3.71)	0.536	-1.71 (-3.61, 0.19)	0.078	-0.36 (-2.63, 1.90)	0.752
Q3	1.82 (-1.02, 4.66)	0.209	-1.79 (-3.71, 0.12)	0.066	1.08 (-1.90, 4.08)	0.476
Q4	4.28 (1.46, 7.09)	0.003^**^	-1.09 (-2.99, 0.81)	0.259	3.69 (-0.77, 8.17)	0.105
*P* for trend		0.002^**^		0.295		0.146

Crude model adjust for: none

Model I adjust for: age and nationality

Model II adjust for: age, nationality, marital status, high, weight, BMI, DBP, SBP, TC, LDL-C, TG, TP, Alb, Hb, globulin, TB, direct bilirubin, ALT, AST, transglutaminase, ALP, FBG, serum calcium, serum creatinine, UA, serum phosphorus, and GH

^*^P < 0.05, ^**^P < 0.01, ^***^P < 0.001

In contrast, the female cohort exhibited a positive association between HDL-C and lumbar BMD in the unadjusted model (*β* = 4.19, 95% CI 1.01 to 7.36, *P* = 0.009). However, after adjusting for all covariates in Model II, this association became negative (*β* = -4.77, 95% CI -8.63 to -0.90, *P* = 0.015). Following the conversion of HDL-C into quartiles, a significant positive correlation was observed only for participants in Q4 in the unadjusted model.

### Subgroup analysis

Table 6 presents the results of the subgroup analyses. Stratified analyses based on age revealed that the relationship between HDL-C and lumbar BMD was influenced by age exclusively in the male cohort. HDL-C exhibited a negative correlation with lumbar BMD in men aged > 45 (*β* = -7.47, 95% CI -13.22 to -1.72, *P* = 0.010) and < = 45 (*β* = -7.45, 95% CI -11.13 to -3.77, *P* < 0.001). When stratifying by BMI, with cut off values of 24 and 28 kg/m^2^, it was evident that male HDL-C displayed a negative association with lumbar BMD across all BMI strata. Notably, this effect was most pronounced among individuals with a BMI greater than 28 kg/m^2^ (*β* = -12.14, 95% CI -19.18 to -5.10, *P* < 0.001). Conversely, in the female cohort, HDL-C exhibited a significantly negative association with lumbar BMD only within the BMI range of 24–28 kg/m^2^ (*β* = -8.47, 95% CI -15.29 to -1.64, *P* = 0.015).

**Table 6:** HDL-C and lumbar BMD were subgroup analyzed and stratified by age and BMI

Subgroup analysis	β (95%CI)	*P* value	*P* for interaction
**Male**			
Age (year)			0.197
<= 45	-7.47 (-13.22, -1.72)	0.010^*^	
> 45	-7.45 (-11.13, -3.77)	< 0.001^***^	
BMI (kg/m^2^)			1.000
<= 24	-6.36 (-12.18, -0.53)	0.032^*^	
> 24, <= 28	-10.51 (-14.54, -6.47)	< 0.001^***^	
> 28	-12.14 (-19.18, -5.10)	< 0.001^***^	
**Female**			
Age (year)			0.812
<= 45	-2.69 (-12.65, 7.27)	0.596	
> 45	-1.30 (-6.46, 3.86)	0.621	
BMI (kg/m^2^)			0.093
<= 24	-3.23 (-8.38, 1.91)	0.218	
> 24, <= 28	-8.47 (-15.29, -1.64)	0.015^*^	
> 28	-1.20 (-15.07, 12.66)	0.864	

Each stratification was adjusted for all factors (age, nationality, marital status, high, weight, BMI, DBP, SBP, TC, LDL-C, TG, TP, Alb, Hb, globulin, TB, direct bilirubin, ALT, AST, transglutaminase, ALP, FBG, serum calcium, serum creatinine, UA, serum phosphorus, and GH), except for the stratification factor itself

^*^P < 0.05, ^**^P < 0.01, ^***^P < 0.001

### Nonrectilinear relationship analysis

**Fig. 4:**
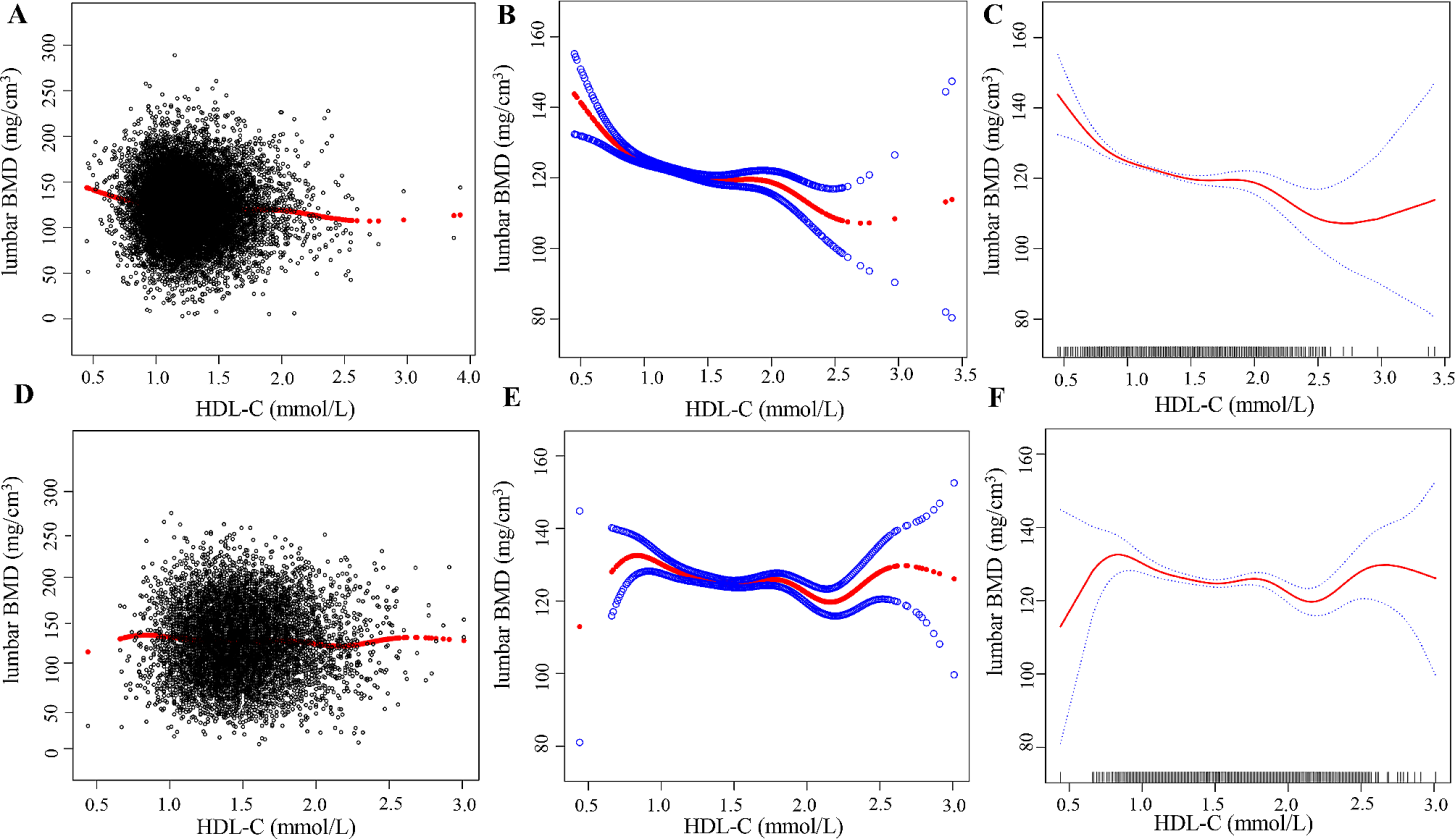
Relationship between HDL-C and lumbar BMD. **A**-**C** for male, **D**-**F** for female. **A** and **D**: Each black hollow point exhibits one participant. **B**, **C**, **E**, and **F**: Solid red line illustrates the fitted smooth curve among variables. Age, nationality, marital status, high, weight, BMI, DBP, SBP, TC, LDL-C, TG, TP, Alb, Hb, globulin, TB, direct bilirubin, ALT, AST, transglutaminase, ALP, FBG, serum calcium, serum creatinine, UA, serum phosphorus, and GH were adjusted

**Fig. 5:**
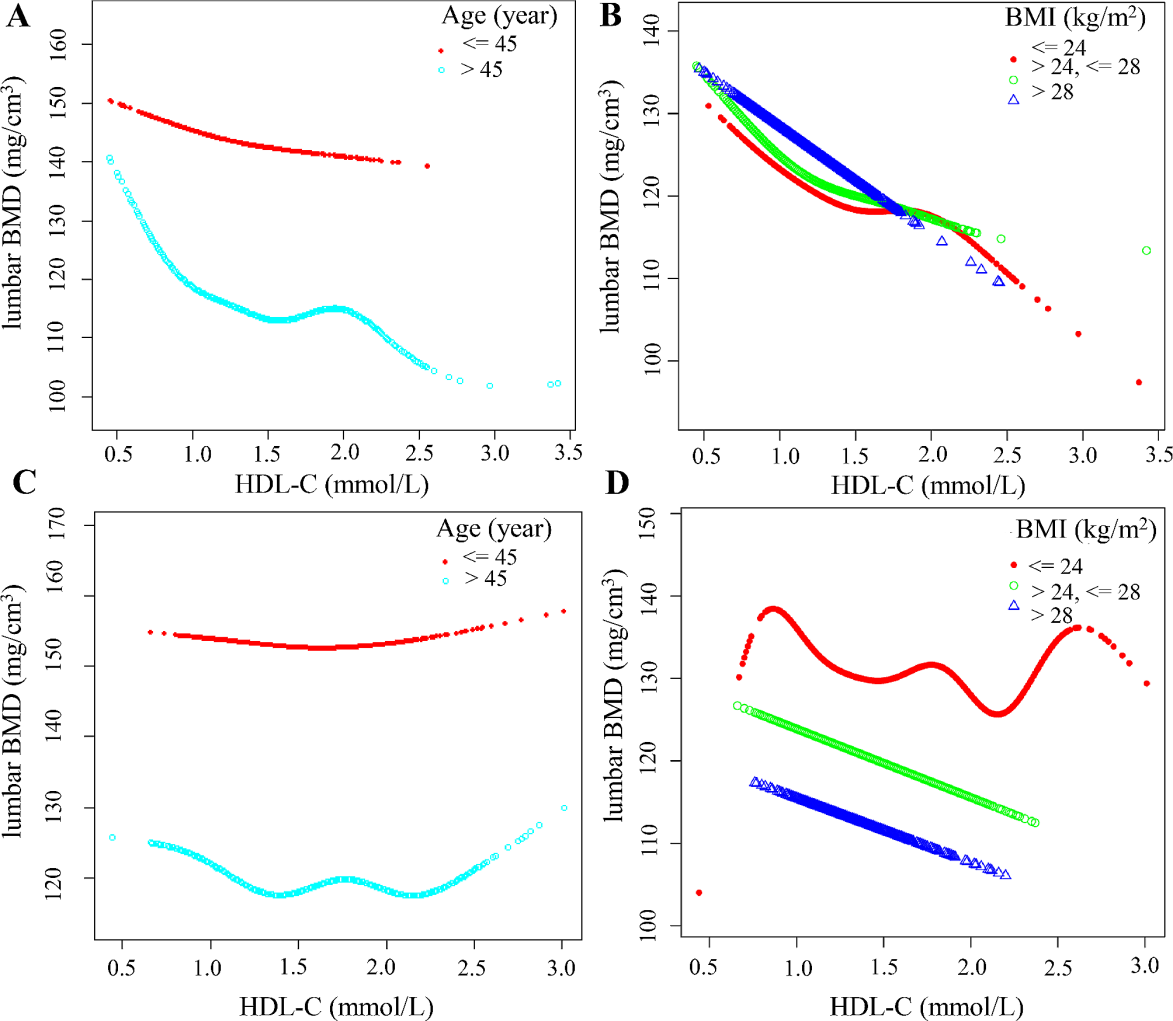
Association between HDL-C and lumbar BMD stratified by tertiles of age and BMI. Nationality, marital status, high, weight, DBP, SBP, TC, LDL-C, TG, TP, Alb, Hb, globulin, TB, direct bilirubin, ALT, AST, transglutaminase, ALP, FBG, serum calcium, serum creatinine, UA, serum phosphorus, and GH were adjusted. **A**-**B** for male, **C**-**D** for female. **A** and **C**: The relationship between HDL-C and lumbar BMD stratified by age. **B** and **D**: The relationship between HDL-C and lumbar BMD stratified by BMI

In this study, the results of age- and BMI-stratified analyses were subjected to segmented linear regression and smoothed curve fitting (Table 7; Fig. 4). In the male cohort, a negative effect of HDL-C on lumbar BMD was observed, both in individuals under 45 years of age (HDL-C inflection point: 0.86 mmol/L) and those aged 45 or older (HDL-C inflection point: 1.12 mmol/L). Notably, the most pronounced negative effect occurred to the left of the inflection point (Fig. 5A). The greatest negative effect was observed when BMI was within the range of 24–28 kg/m^2^, and HDL-C was to the left of the inflection point (1.09 mmol/L) (*β* = -24.60, 95% CI -35.10 to -14.11) (Fig. 5B). Conversely, in women aged over 45, a segmented U-shaped relationship was identified between HDL-C and lumbar BMD, with an inflection point at 1.35 mmol/L for HDL-C. To the left of the inflection point, a negative correlation was observed, while to the right of the inflection point, a positive correlation was noted (Fig. 5C). Among women, the most significant negative effect of HDL-C on lumbar BMD was observed within the BMI range of 24–28 kg/m^2^ (*β* = -8.47, 95% CI -15.29 to -1.64) (Fig. 5D).

**Table 7:** Multivariate regression analysis of the effect of HDL-C on BMD in different gender populations

	Linear regression	Break point (K)	< K	> K	LLR test
	β (95%CI)		β (95%CI)	β (95%CI)	P
**Male**					
Age (year)					
<= 45	-7.47 (-13.22, -1.72)	0.86	-55.88 (-101.14, -10.62)	-6.05 (-11.94, -0.15)	0.034^*^
> 45	-7.45 (-11.13, -3.77)	1.12	-24.74 (-33.76, -15.72)	-3.97 (-8.00, 0.06)	< 0.001^***^
BMI (kg/m^2^)					
<= 24	-6.36 (-12.18, -0.53)	1.28	-15.56 (-25.64, -5.49)	-2.83 (-9.45, 3.79)	0.028^*^
> 24, <= 28	-10.51 (-14.54, -6.47)	1.09	-24.60 (-35.10, -14.11)	-7.43 (-11.98, -2.87)	0.004^**^
> 28	-12.14 (-19.18, -5.10)	1.45	-9.64 (-17.73, -1.55)	-23.58 (-43.14, -4.02)	0.217
**Female**					
Age (year)					
<= 45	-2.69 (-12.65, 7.27)	1.64	-8.44 (-20.42, 3.53)	5.37 (-8.27, 19.02)	0.087
> 45	-1.30 (-6.46, 3.86)	1.35	-13.62 (-23.71, -3.53)	2.85 (-3.07, 8.79)	0.005^**^
BMI (kg/m^2^)					
<= 24	-3.23 (-8.38, 1.91)	1.34	-16.55 (-27.87, -5.24)	0.01 (-5.69, 5.70)	0.009^**^
> 24, <= 28	-8.47 (-15.29, -1.64)	1.83	-5.69 (-13.06, 1.67)	-30.91 (-54.53, -7.28)	0.050
> 28	-1.20 (-15.07, 12.66)	1.02	-52.76 (-139.48, 33.96)	0.21 (-13.84, 14.28)	0.226

Each stratification was adjusted for all factors (age, nationality, marital status, high, weight, BMI, DBP, SBP, TC, LDL-C, TG, TP, Alb, Hb, globulin, TB, direct bilirubin, ALT, AST, transglutaminase, ALP, FBG, serum calcium, serum creatinine, UA, serum phosphorus, and GH), except for the stratification factor itself

^*^P < 0.05, ^**^P < 0.01, ^***^P < 0.001

## Discussion

This study investigated the correlation between HDL-C and lumbar BMD within the Chinese population utilizing a substantial sample of consecutive 5-year data from the same health management center across genders. Upon adjusting for confounding variables, the research findings reveal that higher levels of HDL-C are associated with reduced lumbar BMD in men, regardless of age and BMI. Notably, the detrimental effect of HDL-C on lumbar BMD is most pronounced when BMI exceeds 28 kg/m^2^ and HDL-C levels are above 1.45 mmol/L. Conversely, when BMI falls within the range of 24–28 kg/m^2^, HDL-C exhibits a negative association with lumbar BMD in the female cohort. This study marks the first to demonstrate the gender-specific association between HDL-C and lumbar BMD in a Chinese population without overt health issues.

The interplay between lipid metabolism and bone metabolism has attracted substantial interest. A growing body of biological and epidemiological evidence has underscored the connection between cardiovascular disease and OP, with lipid metabolism serving as an intermediary factor between these two conditions [17]. It is noteworthy that osteoblasts, chondrocytes, and adipocytes all originate from a common precursor cell, mesenchymal stem cells. The direction of differentiation is governed by the Wnt/β-catenin signaling pathway, and when this pathway is obstructed, bone marrow stromal cells tend to differentiate into adipocytes. This mechanism forms the physiological basis for the interdependence of lipid metabolism and bone metabolism [18].

Cholesterol and its metabolites play a pivotal role in regulating osteoblast and osteoclast differentiation and activation, thereby influencing bone homeostasis [19]. HDL-C, as one of the body’s key lipids, is implicated in various metabolic diseases [20] and is known for its role in reducing the incidence of cardiovascular disease through reverse cholesterol transport [21]. Previous studies have suggested a potential link between HDL-C and OP [8]. For instance, Kha et al. [22] reported that certain oxysterols can stimulate osteogenic differentiation of mesenchymal stem cells, and high levels of HDL-C may remove oxysterols from peripheral tissues, thereby negatively affecting osteogenic differentiation. In addition, some of the apolipoproteins contained in HDL-C influence both adipose tissue and bone. An increase in bone marrow adipocytes also deteriorates the osteogenic function of bone, thus affecting the process of osteosynthesis [23]. However, the relationship between HDL-C and BMD remains unclear due to the heterogeneity of studies, stemming from differing study designs, sampling methods, controlled confounding variables, and ethnic distributions that have yet to be harmonized. In this context, investigating the effect of HDL-C on BMD serves as a crucial step toward providing a fresh foundation for OP prevention and treatment, as well as offering guidance to individuals with high HDL-C levels to mitigate OP risk.

After adjusting for covariates, this investigation revealed a correlation between elevated HDL-C levels and reduced lumbar BMD within the male cohort, as observed in the multiple linear regression model. This negative correlation was most pronounced when BMI > 28 kg/m^2^ and HDL-C > 1.45 mmol/L. Previous research has established BMI as a critical intermediary variable when examining the connection between lipids and BMD [24]. Obesity, typically assessed by BMI, frequently intersects with lipid metabolism, resulting in decreased HDL-C levels [25]. Moreover, there exists a positive correlation between BMI and BMD [26]. Consequently, elevated HDL-C levels in obese males frequently lead to diminished BMD. Cui et al. observed a higher incidence of OP in individuals with a BMI < 18.5 kg/m^2^ than in those with a BMI ≥ 18.5 kg/m^2^, irrespective of sex [27]. Alternatively, the inverse relationship between BMD and HDL-C concentration could be attributed to reduced oxysterol availability at heightened HDL-C levels, which could impede the osteogenic differentiation process of mesenchymal stem cells [22]. A study conducted by Yang et al., involving 1,040 Chinese men, unveiled a negative correlation between total lumbar BMD and HDL-C levels [28]. These findings align with the conclusions drawn by Kuipers et al. [29] and Choi et al. [30]. Furthermore, an analysis including men aged 20 to 59 based on the 2011–2018 National Health and Nutrition Examination Survey (NHANES) reported a U-shaped relationship between HDL-C and lumbar BMD in males [8]. Another noteworthy result of this investigation is the persistence of the inverse association between HDL-C and BMD in age-stratified analyses, with 45 years as the pivotal point. This suggests that age exerts minimal influence on BMD alterations in males. This suggests that estrogen may mediate the relationship between HDL-C and lumbar BMD, and estrogen plays an important role in the regulation of lipid metabolism and bone resorption. Importantly, estrogen fluctuations are less pronounced in males throughout their lifespan [31]. Hence, the influence of age on BMD is deemed insignificant.

The most intriguing findings within the female cohort of this study indicate that, after adjusting for confounding factors, multiple linear regression models demonstrate an adverse correlation between elevated HDL-C levels and reduced lumbar BMD levels. Stratified analyses unveiled a significant negative correlation between HDL-C and lumbar BMD within the BMI range of 24–28 kg/m^2^. Nevertheless, when considering the stratified variable of probable age at menopause (45 years) in the analysis, no discernible correlation emerged between HDL-C and lumbar BMD. Zhang et al. conducted a cross-sectional study examining the association between lipid profiles and BMD in approximately 1,116 Chinese women aged approximately 30 years. Their findings revealed a curvilinear relationship between HDL-C and lumbar BMD, characterized by a negative correlation on the left side of the inflection point, which was identified at 2.37 mmol/L [32]. A substantial dataset study from China identified higher levels of HDL-C as an independent risk factor for OP; however, it noted that increasing age exerts a more substantial adverse effect on BMD in women [13]. Adami et al. investigated a cohort of 481 women, aged 68–75 years, recruited from the community, and observed a negative association between their lumbar BMD and HDL-C levels [33]. Conversely, data based on 958 postmenopausal Korean women exhibited no significant association between serum lipid profiles, including HDL-C, and BMD [34]. Similarly, another study involving 170 women aged 50–70 years in Iran also reported no significant correlation between HDL-C and BMD [35]. While prior research has indicated that the use of hormone replacement therapy in postmenopausal women is linked to elevated HDL-C and BMD levels [36], the present study’s data did not provide support for this association, possibly due to the hereditary characteristics shared by HDL-C and BMD [37]. In contrast, Xie’s study reported a positive association between HDL-C and lumbar BMD in American women aged 20 to 59 years [8].

Divergent conclusions regarding the association between HDL-C and lumbar BMD in women may be attributed to variations in researchers’ choice of study variables. Additionally, interpreting this relationship in the female population is challenging due to their unique physiological attributes. Notably, the advantage of a larger sample size in the current study lends greater credibility to the evidence regarding the association between HDL-C and lumbar BMD. However, the causal relationship between HDL-C and lumbar BMD cannot be known in this study, which is also the direction of our future research.

### Study strengths and limitations

In contrast to preceding investigations, this study elected to include individuals categorized as apparently healthy, rendering the findings notably applicable to the broader population. Additionally, the study’s substantial sample size facilitated subgroup analyses based on age and BMI within diverse gender cohorts. While the current study diligently considered and effectively adjusted for confounding factors, certain limitations persist: First, the inherent constraints of cross-sectional studies preclude the establishment of a causal relationship between HDL-C and lumbar BMD in this investigation. Second, the unavailability of endocrine indicators, including sex hormones, for most participants can be attributed to variations in the participants’ health screening programs. Consequently, this facet of the results could not be integrated into the covariate analysis. Last, it is worth noting that the study’s reliance on a sample derived solely from a single health management center resulted in a high degree of homogeneity within the sample. However, this homogeneity diminishes the generalizability of the study’s conclusions. Therefore, it is advisable to corroborate the findings of this study through comparative analyses conducted in a multicenter health management context.

## Conclusion

Our study has identified that elevated levels of HDL-C can influence bone loss in a Chinese population aged 20 to 80 years. This decrease in BMD was particularly prominent among obese men. Furthermore, women with a BMI ranging from 24 to 28 kg/m^2^ also exhibited a significant reduction in BMD. Consequently, individuals with elevated HDL-C levels should undergo regular clinical monitoring to reduce the risk of OP.

## Data Availability

No datasets were generated or analysed during the current study.
